# A collaborative, academic approach to optimizing the national clinical research infrastructure: The first year of the Trial Innovation Network

**DOI:** 10.1017/cts.2018.319

**Published:** 2018-11-27

**Authors:** Gordon R. Bernard, Paul A. Harris, Jill M. Pulley, Daniel K. Benjamin, Jonathan Michael Dean, Daniel E. Ford, Daniel F. Hanley, Harry P. Selker, Consuelo H. Wilkins

**Affiliations:** 1 Vanderbilt Institute for Clinical and Translational Research, Vanderbilt University Medical Center, Nashville, TN, USA; 2 Duke Clinical Research Institute, Duke University Medical Center, Durham, NC, USA; 3 Department of Pediatrics, University of Utah School of Medicine, Salt Lake City, UT, USA; 4 Institute for Clinical and Translational Research, Johns Hopkins School of Medicine, Baltimore, MD, USA; 5 Acute Care Neurology, Johns Hopkins University, Baltimore, MD, USA; 6 Institute for Clinical Research and Health Policy Studies, Tufts Medical Center, Boston, MA, USA; 7 Department of Medicine, Vanderbilt University Medical Center and Department of Internal Medicine, Meharry Medical College, Nashville, TN, USA

**Keywords:** Trial Innovation Network, clinical trials, CTSA program, multisite clinical research, innovation

## Abstract

Inefficiencies in the national clinical research infrastructure have been apparent for decades. The National Center for Advancing Translational Science—sponsored Clinical and Translational Science Award (CTSA) program is able to address such inefficiencies. The Trial Innovation Network (TIN) is a collaborative initiative with the CTSA program and other National Institutes of Health (NIH) Institutes and Centers that addresses critical roadblocks to accelerate the translation of novel interventions to clinical practice. The TIN’s mission is to execute high-quality trials in a quick, cost-efficient manner. The TIN awardees are composed of 3 Trial Innovation Centers, the Recruitment Innovation Center, and the individual CTSA institutions that have identified TIN Liaison units. The TIN has launched a national scale single (central) Institutional Review Board system, master contracting agreements, quality-by-design approaches, novel recruitment support methods, and applies evidence-based strategies to recruitment and patient engagement. The TIN has received 113 submissions from 39 different CTSA institutions and 8 non-CTSA Institutions, with projects associated with 12 different NIH Institutes and Centers across a wide range of clinical/disease areas. Already more than 150 unique health systems/organizations are involved as sites in TIN-related multisite studies. The TIN will begin to capture data and metrics that quantify increased efficiency and quality improvement during operations.

## Introduction

Clinical trials are costly and challenging to complete. A recent analysis found that the average cost to develop and gain marketing approval for a drug is $2.56 billion [1]; a single phase of a clinical trial can range from $22.2 to $71.3 million, depending on therapeutic area [2] and worse, most fail. In addition to the financial barriers for conducting clinical trials, other hurdles include inefficiencies in developing a study design, duplicative review processes (e.g., Institutional Review Board), nonstandard contracting language, recruitment challenges, inefficient study site selection and training and cumbersome research data monitoring. Problems extend beyond study completion; approximately half of the results across many types of studies published in biomedical journals are overstated or are altogether incorrect [3]. An overhaul of the way clinical trials are designed and conducted is essential if the academic research infrastructure is to increase its impact on health and healthcare.

### The Early Clinical and Translational Science Award (CTSA) Program

The CTSA program’s goal in 2006 (under the National Center for Research Resources) was to propel institutions to create a “*novel, and integrative academic home for clinical and translational science*.” In Dr Elias Zerhouni’s early and pioneering vision, CTSAs were intended to advance the assembly of institutional academic “homes” that can provide integrated intellectual and physical resources for the conduct of original clinical and translational science [4]. Thus, during the first funding cycles, CTSAs were established across the country and served as the singular homes for translational research at their respective institutions. Dr Zerhouni also envisioned that the assembled consortium of CTSA sites would provide a research environment that is more nimble, conducive to, and responsive to the demands of modern translational and clinical research [4]. The early CTSAs did just that, and transformed an ineffective clinical and translational science infrastructure through 2 primary methods: development of metrics to assess the length of the processes at individual institutions, and by creating common approaches to contracts and shared Institutional Review Board (IRB) considerations, thereby avoiding redundancy in multicenter studies and accelerating the start-up phase of clinical research [5]. Strategic goals were subsequently formalized which included enhancing the national capability for clinical and translational research [6]. Consortium-wide efforts around recruitment, good clinical practice qualifications, competencies and training, data management, IRB streamlining and contract streamlining ensued; these were collaborative initiatives led by CTSA program investigators that supported the formation of, and are now being leveraged in and tested by, the newly formalized “Trial Innovation Network (TIN)” [7].

### The New CTSA TIN

The TIN is a new collaborative initiative within the National Center for Advancing Translational Science (NCATS) CTSA program that leveraged the prior work of the CTSA program and brought in new capability from clinical trial intensive groups. Clinical trialists with decades of experience in conducting large-scale, multisite regional, national, and international clinical trials that recruit and engage challenging populations comprise TIN leadership. Through innovation, the network will address critical roadblocks in clinical trials and accelerate the translation of novel discoveries and interventions in real-world clinical practice. For example, in clinical trials, numerous approvals must be in place before patients can be enrolled; removing one bottleneck (e.g., IRB review) simply shifts the bottleneck to another process. The novelty of the TIN is to synchronously address all potential bottlenecks by systematizing and streamlining approvals and processes. Through experience gained by conducting hundreds of trials, we have identified these bottlenecks and developed approaches to streamline all approvals to achieve maximally efficient study initiation, including confidentiality agreements, sponsor contracts and site subcontracts, trial registration, protocol finalization, investigator meetings, Data & Safety Monitoring Board operations, Investigational New Drug Application/Investigational Device Exemption, budgets, and IRB review. The TIN is also unique in its ability to “right-size a Network” for any given proposal, rather than relying on pre-identified existing networks. The ultimate mission of the TIN is to execute highest quality trials more quickly, and in a cost-efficient manner. The TIN awardees are composed of 3 key organizational partners: 3 Trial Innovation Centers (TICs), the Recruitment Innovation Center (RIC), and the individual CTSA institutions that have identified TIN Liaison units. Short-term and long-term key metrics for the TIN are still being developed and refined, but the focus is directed on decreasing complexity, increasing efficiency and productivity, and accelerating translational research to get new treatments to patients faster.

#### TICs

The TICs develop, demonstrate, and disseminate innovative ways to increase the quality and efficiency of multisite clinical research. The TICs provide collaborative support for a broad range of multisite clinical studies that include trials across the human lifespan, trials for diagnostic testing or development of therapeutics such as drugs, biologics, and devices, as well as behavioral interventions. TICs also are actively exploring innovative approaches aimed at streamlining trial implementation, promoting high-quality multisite trials, and the dissemination of successful advances in process and approach. The TICs are charged with supporting Central IRB (CIRB), contracting, regulatory, protocol, and consent development as well as performance and quality monitoring. The 3 TICs (Utah, Duke/Vanderbilt, and Johns Hopkins/Tufts) are all experienced in supporting these activities.

#### RIC

The RIC strives to increase the likelihood of success for multisite clinical trials by developing informatics-driven approaches to assess the site-specific availability of potential participants during the trial planning and by developing community-driven approaches to participant recruitment and retention. The RIC is already achieving these expectations by optimizing electronic health record (EHR) and cohort discovery tools using a multi-modal approach to enable broad participation in site feasibility assessments, conducting targeted community engagement studios [8] and other service lines designed to promote recruitment, retention and return of value to participants. The RIC is also involved in building relationships with external stakeholders, establishing a diverse Community Advisory Board as well as creating resources to educate and train local recruitment specialists. The RIC (at Vanderbilt) is experienced in these activities.

#### CTSAs Within the TIN

Each CTSA has—or soon will have—an established group dedicated to the activities associated with the TIN. Although the resources for this function vary by CTSA size, the roles for the TIN Liaisons at each CTSA are the same. These TIN Liaisons include staff with legal, IRB, budgeting, or project management expertise to work with TIC activities, and staff with data, informatics, analysis, privacy protection, study recruitment, communications, or project management expertise to work with the planned RIC activities. The multidisciplinary TIN Liaisons will lead scientific, training, and implementation aspects of the TIN. They will use their experience and knowledge of the local environment to innovatively operationalize the Network at their Institutions, tailoring general network plans into more specific action plans best suited for their sites. In addition, the named liaisons will encourage investigators at their Institutions to generate ideas for multicenter trials and studies that encompass a wide range of disciplines, provide input before protocol implementation, encourage investigators to take advantage of existing CTSA resources, and recognize the essential contributions and efforts of their local teams in executing multicenter clinical trials. Local CTSA PIs will provide a first review and approval of any proposals from their Institution to the TIN. Local resources to enhance study design and recruitment will continue to benefit investigators. CTSA PIs will be included on initial TIN communications with investigators to ensure a collaborative relationship between the investigator, the CTSA, and the TICs and RIC. Although CTSA institutions are the frontline of the TIN, the TIN will engage many types of additional sites including non-CTSA academic institutions, community hospitals, physician practices, and others.

## Results to Date: Year One

### TIN Resources

Structurally, the TIN features a national scale single (central) IRB system, master contracting agreements, quality-by-design approaches, novel recruitment support methods, and a focus on evidence-based strategies to recruitment and patient engagement, as well as other components critical to trial conduct. These offerings are shared and set in place, meaning they do not need to be recreated for future studies. In addition, the TIN has established its decision-making process and communication format as well as all policies, procedures, staff support, and manuals of operation. Its core resources/services are in place (online Supplementary Appendices 1 and 2). Notably, these procedures have been harmonized among long-standing and large-scale academic clinical research organizations such as Duke Clinical Research Institute, Utah and Hopkins.

**Fig. 1:**
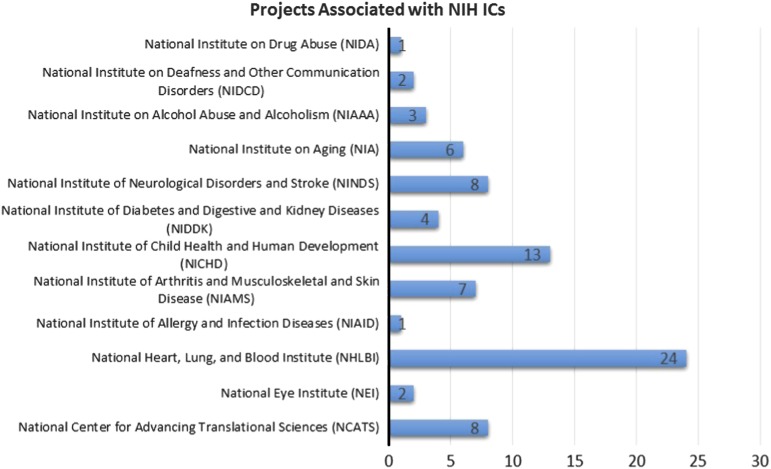
National Institutes of Health (NIH) Institutes and Centers (ICs). Multiple NIH ICs are associated with studies using the Trial Innovation Network.

The TIN provides support and resources in a multi-pronged approach. Specifically, study investigators can request an initial consultation with the TIN or specific services depending on the funding status of the proposal. Unlike some other research networks, the TIN supports clinical trial teams who have been funded by a sponsor other than the TIN for the conduct of the trial. Projects that are already funded or are pending funding may request discrete services (online Supplementary Appendix 1). Projects that are early in the development phase request an initial consultation (online Supplementary Appendix 2). These initial consultations usually consist of several scheduled meetings (in person or web conferences) to discuss applicable topics such as study design, budget, CIRB, timelines, recruitment and retention, study feasibility, study design support, and statistical analysis support. Given that poorly designed studies can place participants at risk with little likelihood of providing an answer to the central question of the trial, an important emphasis of the TIN is optimized study quality. Recognizing that that national clinical research infrastructure must include and embrace performance sites more broadly, already more than *150 unique health systems/organizations* are involved as sites in TIN-related multisite studies. There are 12 different NIH Institutes and Centers (ICs) involved (see Fig. 1).

NIH IC involvement has been in several different ways. NIH ICs are often introduced to the TIN via the many NCATS presentations and training opportunities. In addition, once a project is submitted to the TIN, the NIH IC PO is often invited to attend the consultations and conversations whenever appropriate and agreed to by the involved parties. This inclusive approach has been an effective technique in facilitating dialogs and embracing opportunities for synergy within the project and other prospects associated with the funding agency. In addition, individual investigators with connections to the CTSA Consortium and access to the TIN approach ICs independently, and then a joint process can ensue.

### TIN Innovation

One of the defining features of the TIN is the charge to embrace and apply *innovation* in all activities by leveraging the network as a learning laboratory. The TIN is charged with using novel concepts, approaches and methodologies directed at improving the quality, safety, efficiency, and timely completion of multisite clinical trials. Some innovative opportunities underway within the TIN include:

- Creative use of EHR data to deliver more accurate counts of eligible patients.
- Involving under-represented/minority groups in research design/development of materials to determine impact on enrollment and retention more broadly.
- Testing training approaches for researchers who seek to engage under-represented and marginalized communities.
- Creating a medical device-based cohort participant monitoring/retention system.
- Formalizing and testing e-consent models.
- Assessing whether early budget consultation through the TIN results in the more accurate prediction of site costs.
- Testing a new clinical trial model to streamline the process for bringing on clinical trial sites within a 90-Day cycle.
- Assessing whether an in-person site initiation visit results in improved protocol adherence and trial efficiency compared to a remote site initiation visit.
- Establishing evidence to support a common activity in studies such as site initiation visits.
- Establishing a service that supports efficacy to effectiveness trial designs that works with the investigator and study team to design a study that includes seamless transition from an initial efficacy trial component to a generalizable effectiveness trial component [9].
- Conducting clinical trials within a registry population.
- Service lines designed to support the workflow of leveraging clinical systems.
- Ensuring the use of sophisticated methods such as adaptive trial design, sequential studies, futility analyses through simulation, early termination of trials using frequentist pre-specified or unlimited Bayesian interim analyses for the quality of the studies and the increased protection of human subjects.

### TIN Communications and Coordination

The TIN has created a web portal (https://trialinnovationnetwork.org/) to support communication for the multiple individuals and sites who have a role in the program. The general method of providing resources, best practices and webinars is gaining traction among users through the Web site. Currently ~300 users signed up to be notified of new updates and webinars. Communications resources include a RIC toolkit, Trials Today, newsletters such as the RIC Download, and frequent recruitment webinars, all designed to share best practices and enhance the sustainability of recruitment initiatives at local sites. The TIN CIRB provides resources, tools, and a fully functional web-based platform (https://trialinnovationnetwork.org/smart-irb-exchange/) to operationalize and coordinate the CIRBs activities.

The TIN offers frequent education and training opportunities via its collaboration webinars and liaison-network meetings. Recognizing the need to support the CTSA site liaisons, the TIN holds monthly meetings to discuss current topics, identify needs and describe lessons learned. In addition, there are monthly “Open Forums” focused on various topics that allow the liaisons and CTSA researchers to ask questions in an informal, open-dialog session. A smaller working group with members from 11 institutions has been identified and have volunteered to partner with other liaison members across the CTSA to help champion the TIN and address identified gaps and concerns. These members serve as Peer Advisors to Liaison Team members across the consortium to provide access to sharing local barriers and challenges as well as best practices, beneficial materials, and resources.

### TIN “Hardwiring” Through Legal Agreements

Clinical trial contracting is a major barrier to clinical trial start-up [10]. Data show that an average negotiation of contract terms of 55 days (exclusive of budget and IRB approval) could be reduced to 22 days if a “master agreement” was used [11]. Several years ago this barrier prompted CTSAs to develop a standardized contract that could help reduce delays in trial start-up. With support from NCATS, the Accelerated Clinical Trial Agreement was developed by 25 CTSAs in collaboration with the University Industry Demonstration Partnership, and with input from several pharmaceutical companies. Leaders from the institutions’ contracts and legal offices volunteered for consensus building work over 2 years. Over 300 unique organizations, including a variety of institution types as well as most CTSAs, have legal sign-on acceptance of the terms of the Accelerated Clinical Trial Agreement. CTSAs then developed an adoption of the Federal Demonstration Partnership Clinical Trials Subaward Agreement (FDP-CTSA) *for NIH sponsored trials*. The FDP-CTSA was recently accepted, with minor updates, for use by the TIN. A total of 73 institutions have signed on to the FDP-CTSA. The contract is now being used in 4 TIN studies and data are being collected to determine the rate of acceleration to study start-up.

### Decisions Made

**Fig. 2:**
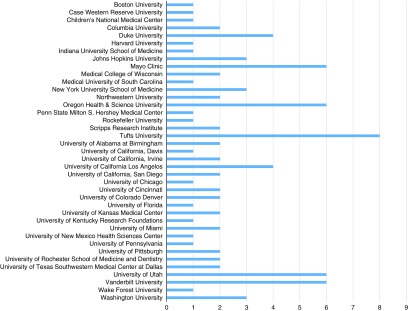
Stakeholder engagement. As of May 18, the Network has received proposals from 39 different Clinical and Translational Science Award institutions. Note: These numbers do not include the pilot testing/demonstration projects (total of 11) conducted among the Trial Innovation Centers sites as systems and services were being established.

**Fig. 3:**
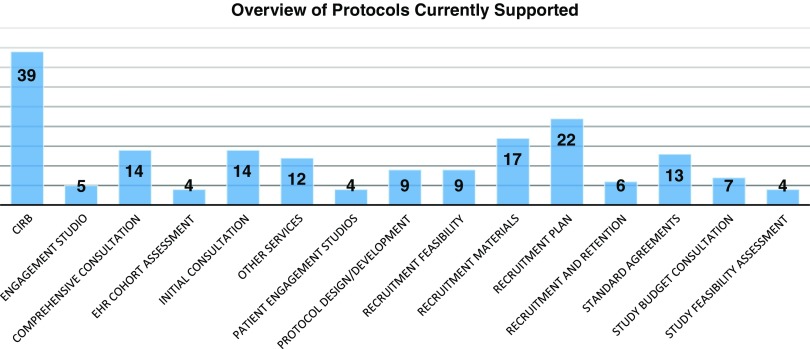
Landscape of resources supporting projects. A range of services is represented in the support provided to Trial Innovation Network studies to date; numbers denote the number of projects receiving each type of service. Each request is counted individually (even for proposals requesting multiple services). CIRB, Central Institutional Review Board.

The TIN has received 113 requests for support from 39 different CTSA institutions and 8 non-CTSA Institutions (see Fig. 2). Requests for TIN infrastructure can be communicated in menu format to be adaptable to researcher needs, depending on the funding status of the proposal. Requests are assessed on use of the TIN infrastructure and scientific merit of each protocol as follows: importance of the question to be addressed; merit of experimental design, including appropriate controls; and safety and welfare of participants. Requesting these services and consults involves submitting an application through the TIN intake system. The system is a web-based, logic flow tool that is designed to only require responses to questions necessary for the types of projects and needs a researcher might consider. Once a proposal is submitted, a member of the Proposal Assessment Team assigns the proposal to a TIC and/or RIC. A TIC/RIC project manager will personally contact the researcher within 5 business days of proposal submission. Proposal Assessment Team procedures are in place to rapidly evaluate and provide initial guidance on the use of TIN resources. On average, the TIN teams have initiated efforts on the projects within 2 days of proposal submission. Snapshot of the 98 of 113 projects receiving support to date are included in the online Supplementary Appendix 3. A description of the types of support are described in the online Supplementary Appendices 1 and 2. The landscape of support being provided varies between projects with some applications receiving one element of support with other projects receiving up to 5 (Fig. 3). Support to date represents a diversity of disease areas (online Supplementary Appendix 4). The 15 projects submitted to the TIN did not receive support. These were early submissions that either were not ready for network involvement, had not engaged their CTSA or were considered to be out of scope according to the funding agency.

### Outcomes

**Fig. 4:**
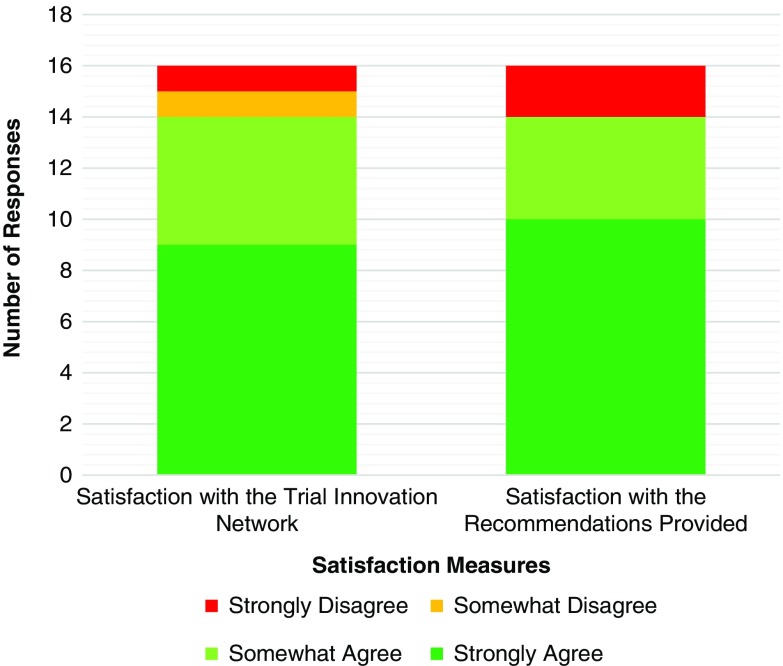
Satisfaction related to the initial consultations. Satisfaction survey responses have been received from 16 study teams.

To date, 59 projects have completed an initial consultation. On average it has taken 71 days from submission to completion of the consultation, with times ranging from 1 to 160 days. Satisfaction surveys have been sent to research teams that have completed an initial consultation with 16 responses to date. Of these, 85% strongly or somewhat agreed that they were satisfied with the overall experience with the TIN. There were 2 respondents who indicated they were not satisfied with their overall experience with the TIN; 1 indicated displeasure with the TIN CIRB’s inability to work with Veterans Affairs (VA) IRBs, which is limited by VA policy. The other respondent reporting dissatisfaction on the Likert scale for this topic also included wholly positive and complimentary questions in the qualitative response opportunities, suggesting that this individual may have incorrectly scored the satisfaction question. See Fig. 4 and online Supplementary Appendix 5 for breakdown of satisfaction results and comments.

## Future Plans and Conclusions

### Budget Tools

One report indicates that budget negotiations and approval lead to up to 49% of study delays [12]. If we could harness the collective expertise of the CTSA institutions to develop tools to improve clinical trial budget creation, the aggregate impact on time and cost savings could be enormous, as studies indicate that sponsors may lose an astonishing $600,000 to $8 million for each day that a trial delays launch [13]. The TIN is in the process of developing a “go/no go” tool that will be able to assist sites in quickly determining budget feasibility. These types of tools will be developed and made freely available for use by institutions collaborating with the TIN.

### Recruitment Infrastructure Support Service

Recruitment challenges continue to plague the national clinical trial system. To solve this problem, the RIC leverages clinical data to support cross-network phenotyping queries to determine site feasibility such that planning numbers can be data driven and realistic. Next innovations will include cataloging and building technical infrastructure models for using the clinical system to improve workflow related to recruitment of participants such as EHR query and alert tools. Tools and processes will also be implemented to obtain input from (potential) participant communities on study inclusion and exclusion criteria for studies. Developing and making available training approaches for researchers to engage under-represented and marginalized communities will help with better engagement of these important groups. Finally, enhanced tools for e-consent will be provided.

### Hardwiring Through Legal Agreements

New efforts will involve other contracting processes including Umbrella Confidentiality Agreements, Data Use Agreements and Material Transfer Agreements. Importantly, these initiatives require the engagement, collaboration, and agreement of all CTSA institutions as well as other major research-oriented institutions to be maximally effective in streamlining and efficiency.

### Increased Awareness and Utilization by NIH ICs

Some NIH ICs have well-established clinical and data coordinating centers to support multicenter trials. Other, smaller organizations may be lacking in this capability and effort and therefore will be more interested in engaging the TIN. As these engagement opportunities grow, we will create dashboards and mechanisms to enhance transparency regarding study activities such as real-time participant enrollment, procedural completion and milestone achievements that will be paramount for reporting to constituents and stakeholders.

### Further Engagement with TIN Liaisons

The TIN liaisons are integral to the success of the TIN by encouraging faculty and investigators to generate ideas for studies, review submissions to the network and leverage their local sites’ expertise to optimize studies before TIN submission. As part of the “value add” to these members, we are working to create access to a Network Dashboards that enhance transparency and provide a task list for individual study activities. For example, we have developed a network dashboard that displays high-level information about submitted proposals, enables the user to contact the assigned TIC/RIC, and displays progress indicators that allow the user to monitor network support progress (see online Supplementary Appendix 6).

Furthermore, in an effort to centralize the requests the TIN sends out to the CTSA sites, the network has developed additional tabs, such as the EHR Cohort Assessment tab, which allows the TIN Liaison Teams to keep track of the work they are doing. Similar to other tabs, the EHR Cohort Assessment tab allows the user to drill down and see metrics related to the request, and centralizes actions for the user (see online Supplementary Appendix 7).

## Conclusions

The TIN is functional and has begun early operations. To ensure the TIN achieves its full potential, the interest of ICs across NIH in leveraging the infrastructure will be critical, as well as support from other sponsors. Many ICs may be reluctant to adopt the newly available TIN services due to issues related to credibility, lack of awareness, and perception of cost inefficiency. The TIN will only overcome these barriers by providing evidence of the value of this centralized, shared, and non-redundant infrastructure. To that end, the TIN will capture data and metrics that quantify increased efficiency and quality improvement. Fortunately, the TIN itself provides a national laboratory to study, understand, and innovate *the process of* conducting clinical trials in the course of its operations. In this way, the process can be continually improved. The TIN intends to accelerate these metrics over time.

## Supplementary material

For supplementary material accompanying this paper visit https://doi.org/10.1017/cts.2018.319. click here to view supplementary material
