# Supplementary material for: A collaborative, academic approach to optimizing the national clinical research infrastructure: The first year of the Trial Innovation Network
Source: J Clin Transl Sci. 2018 Nov 27;2(4):187–92. doi: 10.1017/cts.2018.319 (PMC6474372; doi:10.1017/cts.2018.319)
Supplement: Supplementary file 1 [file S2059866118003199sup.zip › S2059866118003199sup001.docx]

**Appendix 1. Key services.** Foundational service elements and capabilities within the TIN have been established in Year 1 by the TIN awardees.

| **Services** | **What TIN Customers Can Expect** |
| --- | --- |
| **Operationalize Standard Agreements** | With this service, we will provide recommendations on how to use the FDP-CTSA Standard Agreement. This [Standard Agreement](https://trialinnovationnetwork.org/agreement/?key-element=1605) can be used by each of the participating institutions in your multi-site study. |
| **Operationalize Central IRB** | The TIN has established three Central IRBs (CIRBs), which are based on the NCATS Streamlined, Multisite, Accelerated Resources for Trials (SMART) IRB Authorization Agreement, to provide CIRB services to a funded multi-center clinical trial. For each study, the TIN CIRB provides resources, tools, and a web-based platform ([IRB Reliance Exchange](https://trialinnovationnetwork.org/home-page/smart-irb-exchange/)) to operationalize the CIRB. With this service, we will ensure all site investigators understand the process for initiating the use of a CIRB at their local institution, as well how to submit and report to the CIRB from initial submission to study closeout. |
| **Recruitment Plan** | An effective recruitment plan includes strategies to identify and engage specific population(s) of relevance for a trial including how to communicate and market a study in order to engage potential participants and meet realistic enrollment and retention goals. The recruitment plan service includes providing advice and recommendations on recruitment strategies. Key features of this TIN Service include:   - Comprehensive review of study and existing recruitment plan - Assistance with identifying stakeholders and recruitment partners (such as providers and community organizations), as well as locations for participant recruitment - Guidance on understanding unique needs and preferences of potential participants, and barriers and facilitators to recruitment and retention - Tailored advice and recommendations on the most appropriate recruitment and retention strategies to deploy when engaging participants from any community |
| **Recruitment Feasibility Assessment** | Recruitment feasibility assessment is the process of evaluating the possibility of recruiting an adequate number of participants with the appropriate range of characteristics (such as age, gender, race/ethnicity, and health status) to meet enrollment goals on the projected timelines and cost. The recruitment feasibility assessment considers environmental (such as location, competition, prior success recruiting, potential participant pool) strengths and weakness as well as logistical, motivational and behavioral barriers to recruitment and retention.  Key features of this TIN Service include:   - Comprehensive review of study - Assessment of the likelihood study will meet predefined recruitment and retention goals - Environmental scan and assessment of barriers - Review budget for recruitment and retention - Tailored advice to enhance recruitment feasibility |
| **Recruitment Materials** | Recruitment materials may include any written or verbal communication delivered through a range of multimedia channels and platforms to increase enrollment. Key features of this TIN Service include:   - Reviewing recruitment material needs including any dissemination plans - Providing advice, recommendations and templates in order to improve the recruitment of potential participants for specific studies - Sharing best practices - Tailoring materials to specific populations, when appropriate |
| **Community Engagement Studio**^8^ | A Community Engagement Studio is a consultative method that allows meaningful involvement of diverse groups of stakeholders in the planning and implementation of research. The Studios can be used to facilitate project-specific input including guidance and recommendations on identifying and addressing barriers to participation and how to develop or refine recruitment materials and messages. Key features of this TIN Service include:   - Assessing whether a studio might be appropriate based on the needs of a study and if so, where a studio has the potential to be most valuable - Identifying community and/or patient stakeholders for studio - Convening community engagement studio - Summarizing recommendations on how to develop and refine recruitment plan, study messaging and materials. |
| **EHR-Based Cohort Assessment** | The TIN will help consider ways that Electronic Health Record (EHR) data may be leveraged to inform study design and potential site selection. Investigators can expect expert clinical and technical review of a study’s goal recruitment population and high level assessment of computable phenotyping. Projects may also request and receive support required to organize distribution of phenotype algorithms to potential CTSA institutions and collation of results. |
| **Data Management** | Consultation and potential implementation concerning the most efficient collection of study data. Investigators can expect rigorous discussion about eliminating extraneous “curiosity” data points, and emphasis on the proper risk based monitoring to assure the quality of data required for the primary and secondary endpoints of the study. |
| **Statistical Assistance** | CTSA institutions have statistical resources and expertise; with this service, the TIN will work with these teams to confirm statistical assumptions and calculations, as requested. This may be particularly relevant for preparation and presentation of Data Safety Monitoring Board (DSMB) reports. |
| **Study Design** | Collaborative assistance with statistical design respects the statistical expertise at each collaborating institution. Investigators can expect TIC statistical teams to be complimentary to the PI’s existing resources, and during study implementation (if indicated). TIC statisticians will keep the investigator’s local statistical experts involved with the study. |
| **Efficacy-to-Effectiveness (E2E) Trial Design** | The E2E Team works with investigators and teams to support trials that seamlessly transition from a trail that shows efficacy of a treatment in selected participants and settings to an effectiveness trail that shows impact in the wide span of patients and settings in which the treatment will ultimately be used. The objective is to be sure that effectiveness is formally tested, which often is not the case, but to also minimize the cost and time of the additional trial. Thereby, regulatory requirements are met for marketing approval but also other stakeholders have evidence to support wide use in clinical practice.  Besides the elements of study design and operationalization, an E2E Consultation will allow the study team to think through the evidence required by various stakeholders, and prospectively design a study that will allow regulators, payers, patients, healthcare providers and physicians to make informed decisions and thus maximize the impact of the trial. |
