# Supplementary material for: A collaborative, academic approach to optimizing the national clinical research infrastructure: The first year of the Trial Innovation Network
Source: J Clin Transl Sci. 2018 Nov 27;2(4):187–92. doi: 10.1017/cts.2018.319 (PMC6474372; doi:10.1017/cts.2018.319)
Supplement: Supplementary file 1 [file S2059866118003199sup.zip › S2059866118003199sup002.docx]

**Appendix 5. Respondents’ commentary regarding consultation value.** Respondents provided a number of qualitative comments in response to a query about the most valuable aspect of the initial consultation.

| **What did you find most valuable about the Initial Consultation?** |
| --- |
| Outstanding input from experts across multiple disciplines. |
| I felt the TIC team was committed to helping me and my team improve our project. It seems the tools they can bring to the project re IRB, DCC, CCC could help a lot. |
| Suggestions for outreach to referring doctors Approval of the CIRB |
| The comprehensive approach to addressing our study requirements, the excellent communication, the excellent organization, the high level of expertise, and last but not least, the genuine interest in our study. |
| They clarified some of the challenges in working with a central IRB |
| Statistical consult |
| This is an invaluable resource and a wonderful team. The guidance surrounding planning for a quick start-up was fantastic. |
| The initial consultation increased my understanding of the role of the central IRB as we move forward with my grant application. In addition it was reassuring to interact with such pleasant and thoughtful individuals. It gives me confidence that our work together going forward will progress smoothly. |
| Prompt and helpful staff, they were amazing. |
| The recruitment innovation center's proposal of services/ideas were excellent!! |
| The conciseness and professionalism of those at the TIC. |
| A detailed explanation of the process. |
| Many people together in the same room may make the process more streamlined. |
| Information on recruitment services I was not aware of |
| Defining the specific aims of the project. |
