# Supplementary material for: A collaborative, academic approach to optimizing the national clinical research infrastructure: The first year of the Trial Innovation Network
Source: J Clin Transl Sci. 2018 Nov 27;2(4):187–92. doi: 10.1017/cts.2018.319 (PMC6474372; doi:10.1017/cts.2018.319)
Supplement: Supplementary file 1 [file S2059866118003199sup.zip › S2059866118003199sup006.docx]

**Appendix 2. Overview of initial consultation activities**. Projects that are early in the development phase may request an initial consultation and receive input on any of the described activities.

| **Initial Consultations** | **What Customers Can Expect** |
| --- | --- |
| **Study Design** | The TIN will review submitted study material and discuss the proposed design. The discussions may involve the study goals and aims, the methodology, statistical and regulatory considerations, participant recruitment, schedule of assessments, study interventions, or other components of the study. The goal of these discussions is to help work through potential barriers to successful study completion. |
| **Study Budget** | The TIN will provide recommendation on the budget as it relates to the proposed protocol. At the conclusion of an initial consultation, an estimated budget based on the protocol design will be generated. The budget will include estimates for the overall study budget, site/participant budget, and a recruitment budget, if applicable. |
| **Projected Timelines** | The TIN will provide a recommendation on timelines for submission of the application as well as timelines for the overall conduct of the study. This review includes planning, study start-up, conduct, close-out, and final publication. |
| **Recruitment** | The TIN will assess recruitment strategies. At the conclusion of an initial consultation, an assessment of study recruitment will be provided. |
| **Assessment of Study Feasibility** | The TIN will assess the feasibility of the study being successfully completed within the proposed timelines and budget and note any potential barriers. The TIN will provide recommendation to optimize successful completion of the study within the proposed timelines and budget. |
