# Supplementary material for: A collaborative, academic approach to optimizing the national clinical research infrastructure: The first year of the Trial Innovation Network
Source: J Clin Transl Sci. 2018 Nov 27;2(4):187–92. doi: 10.1017/cts.2018.319 (PMC6474372; doi:10.1017/cts.2018.319)

**Appendix 3. Support provided.** As of May 2018, 113 proposals have been submitted and 98 of those have received or are currently receiving support from the TIN.


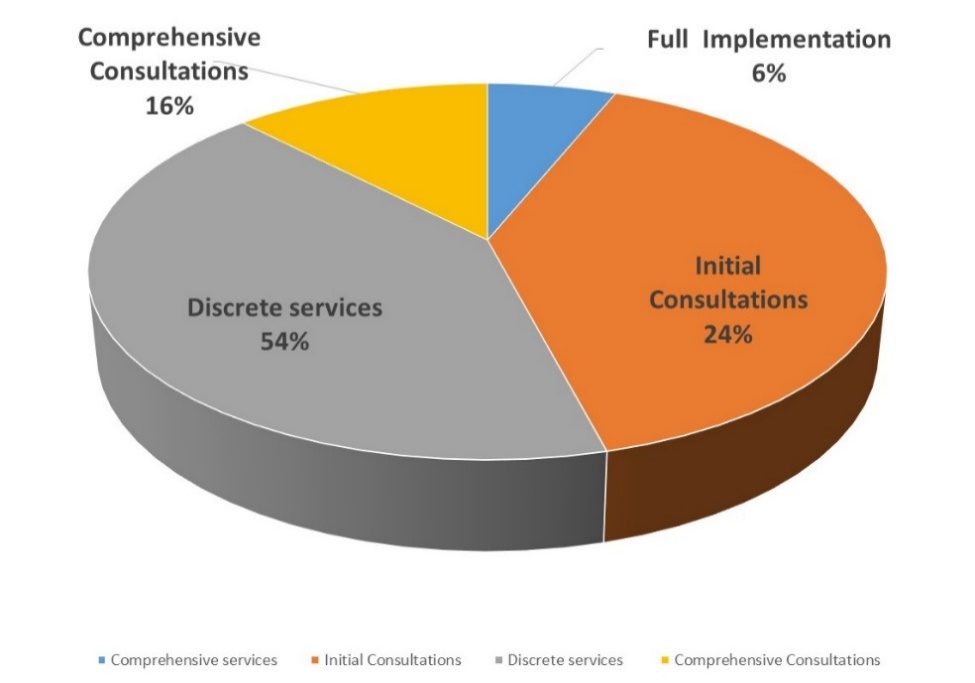

Supplement: Supplementary file 1 [file S2059866118003199sup.zip › S2059866118003199sup007.docx]
